# Supplementary material for: New PEDOT Derivatives Electrocoated on Silicon Nanowires Protected with ALD Nanometric Alumina for Ultrastable Microsupercapacitors
Source: Materials (Basel). 2022 Aug 30;15(17):5997. doi: 10.3390/ma15175997 (PMC9456597; doi:10.3390/ma15175997)
Supplement: Supplementary file 1 [file materials-15-05997-s001.zip › materials-1851891-supplementary.pdf]

Supplementary Materials of:

New PEDOT Derivatives Electrocoated on Silicon Nanowires Protected with ALD Nanometric Alumina for Ultrastable Microsupercapacitors

This section shows results that were not included in the final manuscript, due to their secondary aspect, although they can answer part of readers and reviewers interrogations.

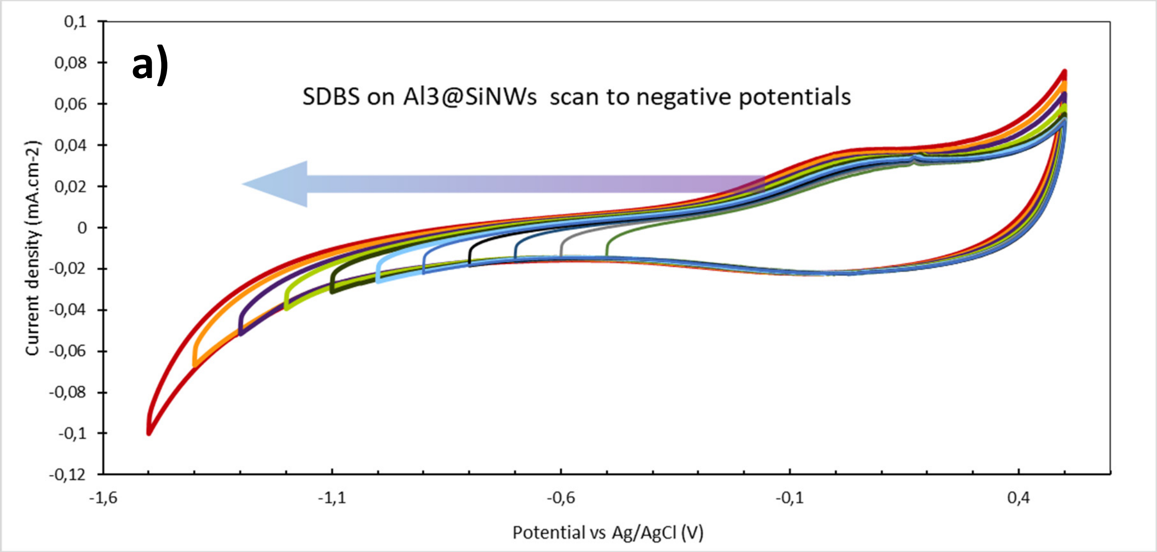

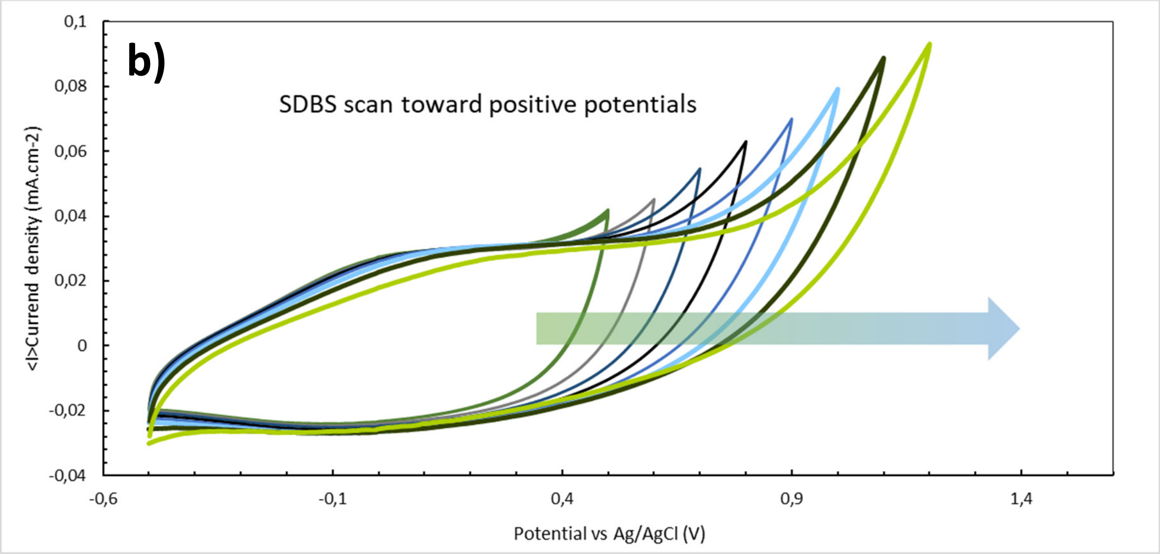

Figure S1 (a,b): Stability Window of PEDOT-OH + Al_3@SiNWs in Na_2SO_4 0.7M, (**a**) for negative and (**b**) positive potential, vs Ag/AgCl. We tried to compromise between the PEDOT electrochemical activity window and the aqueous electrolyte stability, leading us to scanning through -0.9V to 0.9V vs Ag/AgCl

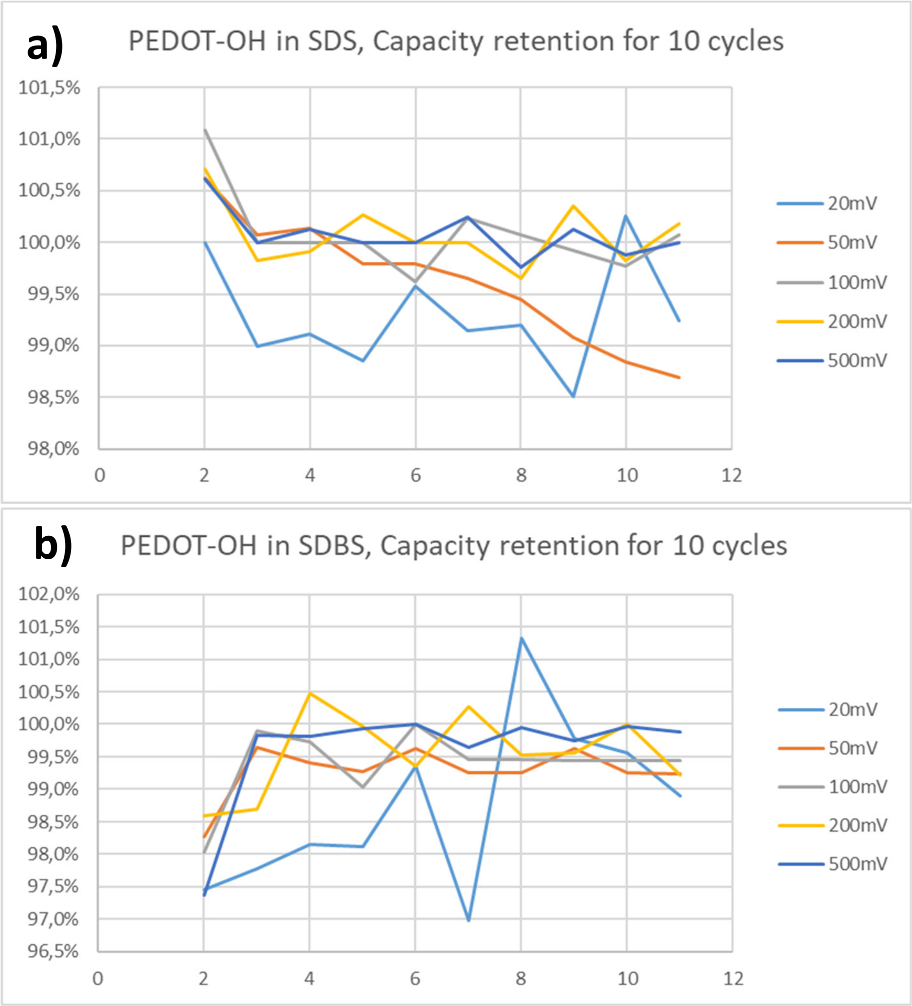

Figure S2 (a,b): Capacity retention through the first cycles for each scan rates, from the cyclic voltammetry of PEDOT-OH + Al_3@SiNWs deposited either by SDS (**a**) or SDBS (**b**). Retention is kept relatively high above 97% for all scan speeds, the 20 mV/s being the only one irregular as they were the starting ones.

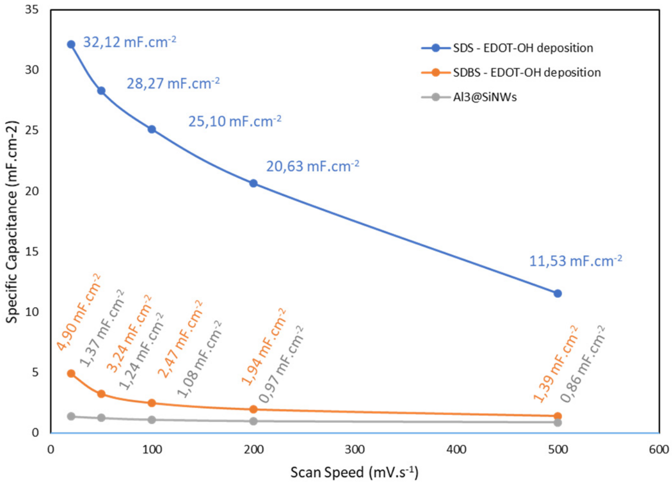

Figure S3: Detail of the capacitances included in the figure 3 of the main text.

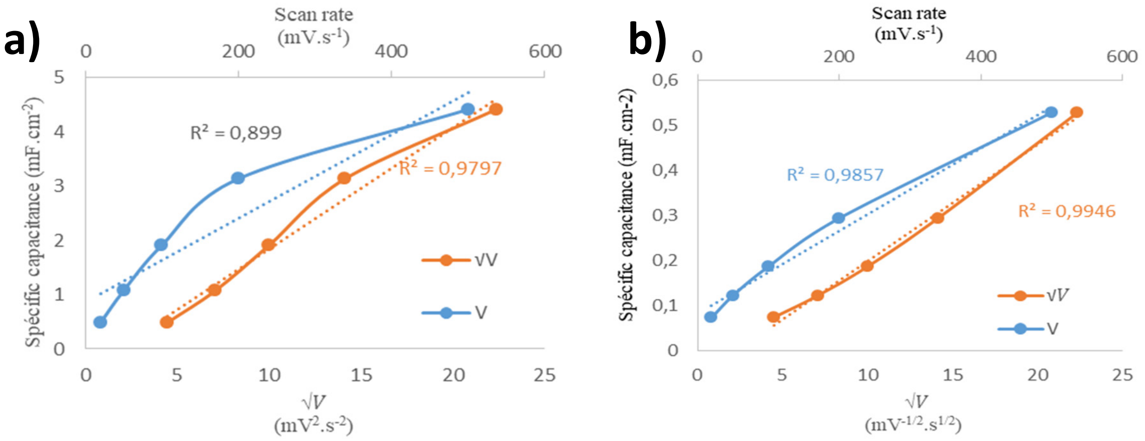

Figure S4 (a,b): Detail of the scan speed effect on the capacitance, this allows us to consider the capacitive or pseudocapacitive behavior of our nanocomposites. Here on the **left SDS** with a square root giving the best fitting of the curve, and on the **right SDBS**, also showing a pseudocapacitive response, following a square root fit

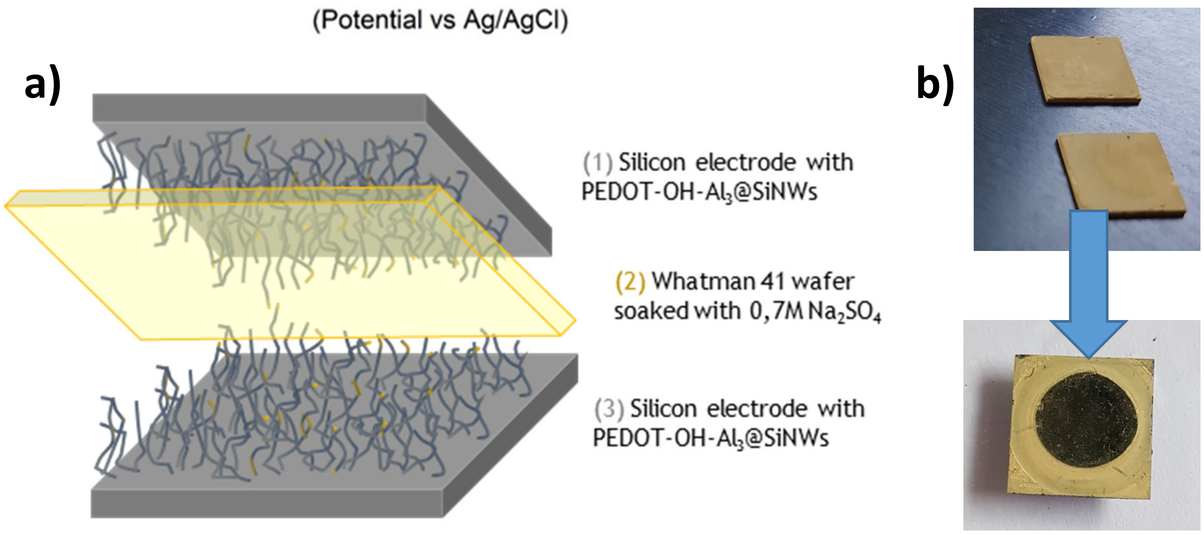

Figure S5 (a,b): (a) Scheme of the all assembly symmetrical cell, with both silicon electrodes each containing the composite PEDOT-OH + Al_3@SiNWs. (b) Images also demonstrate the deposition on the wafer surface after electropolymerization with the darker circular part.

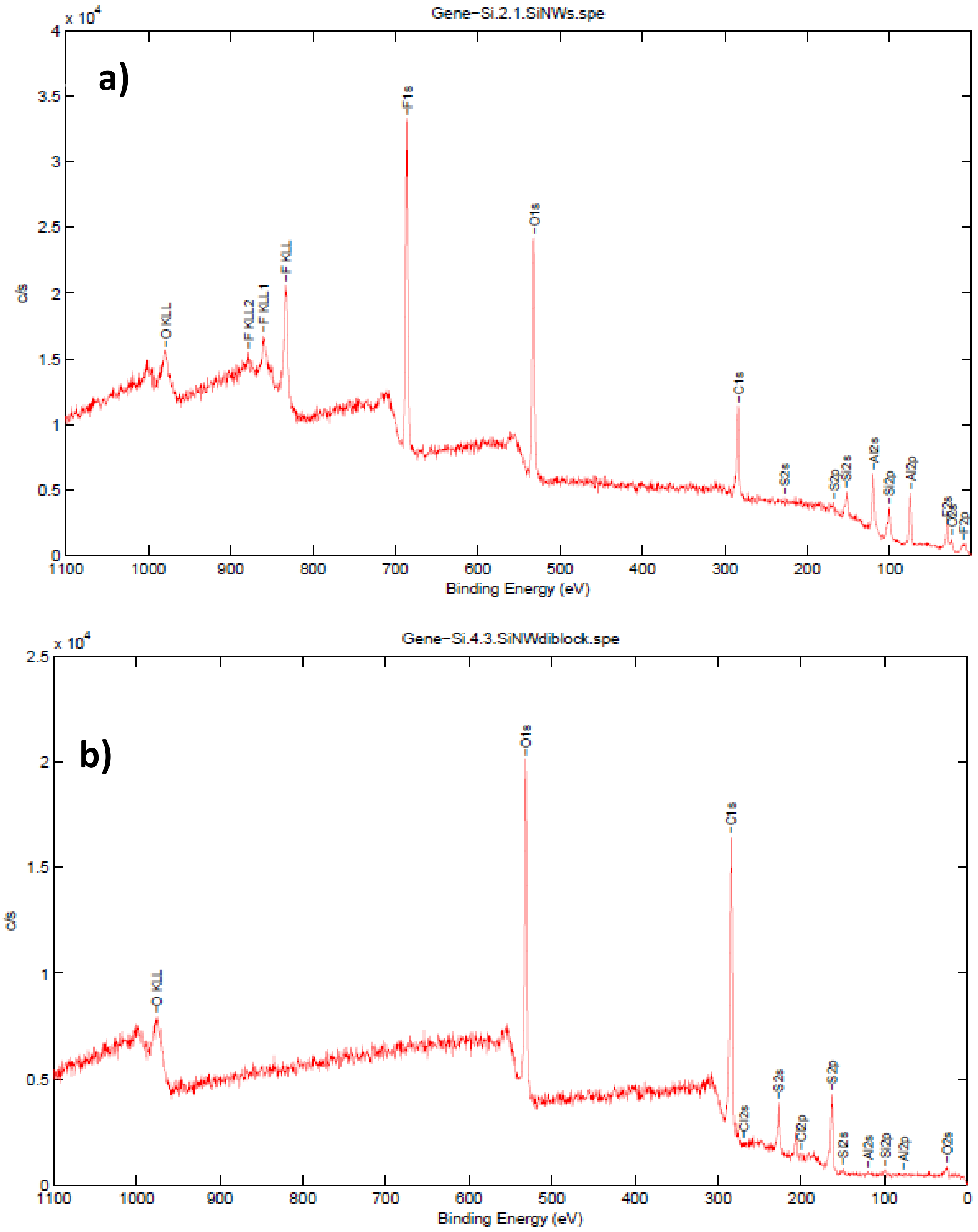

.

Figure S6 (a,b): XPS analysis: Here above (a), we have the signal of the bare Al_3@SiNWs, characterized with Fluor and Aluminum peaks. Bellow (b) we have the composite XPS spectrum. Due to the large amount of polymer deposited after 100 cycles, the signal corresponding to the Al_3@SiNWs is almost invisible, hidden under the polymer thick layer. Mimicking the response of polymer without any nanostructures.
